# Calcium Activation of the Androgen Receptor in Prostate Cells

**DOI:** 10.1155/2023/9907948

**Published:** 2023-12-13

**Authors:** Zeina W. Sharawi, Sawsan M. Khatrawi, Qiaochu Wang, Hongzhao Zhou, Kedra Cyrus, Gai Yan, Becky Hoxter, Bassem R. Haddad, Mary Beth Martin

**Affiliations:** ^1^Departments of Oncology, Georgetown University, Washington, DC 20007, USA; ^2^Department of Genetics and Human Genetics, Howard University, Washington, DC 20059, USA; ^3^Biological Sciences Department, Faculty of Sciences, King AbdulAziz University, Jeddah, Saudi Arabia; ^4^Departments of Biochemistry, Molecular and Cellular Biology, Georgetown University, Washington, DC 20007, USA; ^5^Lombardi Comprehensive Cancer Center, Research Building, 3970 Reservoir Road NW, Washington, DC 20007, USA

## Abstract

**Background:**

Although prostate cancer patients initially respond to androgen deprivation therapy, most patients progress to a resistant phenotype. Castration resistance is due, in part, to intratumoral and/or adrenal synthesis of androgens, overexpression or mutation of the androgen receptor (AR), stabilization of AR by chaperones, and ligand-independent activation of AR. Increasing evidence also links disruption of calcium homeostasis to progression of prostate cancer. Our previous study shows that heavy metal cadmium activates the AR through a ligand-independent mechanism. Cadmium mimics calcium in biological systems due to their similar ionic charge and radius. This study determines whether calcium activates AR and whether first- and second-generation antiandrogens block the ability of calcium to activate the receptor.

**Methods:**

The expression of androgen-responsive genes and calcium channels was measured in prostate cells using a quantitative real-time polymerase chain reaction assay. Cell growth was measured.

**Results:**

To ask whether calcium activates AR, prostate cells were treated with calcium in the absence and presence of the first-generation antiandrogens hydroxyflutamide and bicalutamide and the second-generation antiandrogen enzalutamide, and the expression of androgen-responsive genes and cell growth was measured. In the normal PWR-1E cells and HEK293T cells transiently expressing AR, treatment with calcium increased the expression of androgen-responsive genes by approximately 3-fold. The increase was blocked by enzalutamide but was not consistently blocked by the first-generation antiandrogens. In LNCaP cells which contain a mutant AR, treatment with calcium also increased the expression of androgen-responsive genes by approximately 3-fold, and the increase was more effectively blocked by enzalutamide than by hydroxyflutamide or bicalutamide. Treatment with calcium also increased cell growth that was blocked by enzalutamide. To ask whether dysregulation of calcium channels is associated with castration resistance, calcium channels were measured in the normal PWR-1E prostate cells, the hormone-responsive LNCaP cells, and the castration-resistant VCaP and 22RV1 cells. Compared to normal prostate cells, the hormone-responsive and hormone-resistant cells overexpressed several calcium channels.

**Conclusions:**

The results of this study show that calcium activates AR and increases cell growth and that calcium channels are overexpressed in hormone-responsive and hormone-resistant prostate cancer cells. Taken together, the results suggest a novel role of calcium in the castration-resistant phenotype.

## 1. Introduction

Prostate cancer (PCa) is the most common cancer in men in the United States after skin cancer and the second leading cause of cancer deaths after lung cancer. The American Cancer Society estimates that there will be approximately 268,490 new cases and 34,500 deaths in 2022 (SEER, CANCER.ORG). Androgens and the androgen receptor (AR) are required for normal growth and function of the prostate gland and are critical for all stages of prostate cancer development and progression. The central role of androgens and AR in the development and progression of the disease has led to the use of surgical castration and hormone therapies for the treatment of advanced, relapsed, and metastatic prostate cancer.

Hormone therapy includes treatment with androgen antagonists. The first-generation nonsteroidal androgen antagonists include flutamide (Eulexin), bicalutamide (Casodex), and nilutamide (Nilandron). Flutamide was approved for the treatment of prostate cancer in 1989 [1] and formed the structural basis for other nonsteroidal antiandrogens including bicalutamide and the second-generation antiandrogen enzalutamide. Bicalutamide was approved in 1985 and is the best tolerated and often used with a luteinizing hormone-releasing hormone agonist to block androgen synthesis [2]. Bicalutamide has a higher binding affinity for the androgen receptor and a longer half-life than flutamide or nilutamide [1]. However, bicalutamide has partial agonist activity [3]. Although flutamide has a lower affinity than bicalutamide and nilutamide, it also inhibits the CYP17A1 enzyme [4]. However, hydroxyflutamide, which is the active metabolite of flutamide, has weak agonist activity [5]. The second-generation androgen antagonist enzalutamide (Xtandi) is also a nonsteroidal antiandrogen [6] and has greater efficacy than flutamide and bicalutamide. Enzalutamide has a 4-fold higher binding affinity for the androgen receptor and lacks the agonist activity that is found in both bicalutamide and flutamide [7].

Patients with progressive prostate cancer often develop castration-resistant disease primarily due to the reactivation of the androgen receptor signaling pathway. Although the mechanisms that are responsible for resistance are not completely understood, resistance is associated with excessive synthesis of androgen, overexpression of the androgen receptor and its coactivators, activation of the mutated androgen receptor by antiandrogens and steroids, constitutively active AR splice variants lacking the C-terminal domain, and ligand-independent activation of the androgen receptor by growth factor, cytokine, and inflammatory signaling pathways including epidermal growth factor (EGF), insulin-like growth factor-1, and interleukin-6 signaling pathways [8]. We have previously shown that in breast cancer cells, epidermal growth factor and extracellular calcium activate estrogen receptor-alpha (ER*α*) in the absence of estradiol by increasing intracellular calcium which binds to the ligand-binding domain and activates the receptor [9]. In addition to calcium, we have shown that cadmium activates ER*α* and AR in the absence of hormone [10–12]. Cadmium has an ionic charge of +2 and an effective ionic radius that is similar to calcium and thereby mimics the biological effects of calcium [13, 14]. The ability of calcium to activate ER*α* in the absence of estradiol and to mediate the cross talk between epidermal growth factor and the ligand-binding domain of the receptor suggests that calcium is a physiological ligand of ER*α* and may provide an explanation for the ability of heavy metal cadmium to activate ER*α* and AR. Based on the structural similarities and amino acid homologies between the ligand-binding domains of ER*α* and AR [15] and the similarities between calcium and cadmium, we asked whether calcium is a ligand of the androgen receptor and whether first- and second-generation androgen antagonists block the ability of calcium to activate the receptor. The results show that calcium activates the androgen receptor in the absence of androgen and activation is more effectively inhibited by a second-generation antiandrogen.

## 2. Materials and Methods

### 2.1. Cell Culture

The prostate cancer cells LNCaP, 22RV1, and VCaP were acquired from the Tissue Culture and Biobanking Shared Resource, and the normal prostate cells PWR-1E were acquired from Dr. S. Byers's laboratory at Georgetown University. All cell lines were originally obtained from the American Type Culture Collection. The prostate cancer cell lines LNCaP, 22RV1, and VCaP were maintained in an RPMI medium 1640 (Gibco; Life Technologies) containing 10% of fetal bovine serum (FBS) and 1% of sodium pyruvate. The normal prostate cell line PWR-1E was maintained in keratinocyte-serum-free media supplemented with L-glutamine, human recombinant epidermal growth factor, and bovine pituitary extract (Gibco; Life Technologies). Prior to treatment, PWR-1E, LNCaP, and VCaP cells were plated in improved minimum essential media (IMEM)-containing phenol red and 10% FBS serum. At 70% confluence, the media were changed to IMEM phenol red-free, lipoic acid-free media (Crystalgen) containing 5% charcoal-treated calf serum (CCS; Valley Biomedical) for 48 hours. The cells were treated with dihydroxytestosterone (DHT; 5 nM), the synthetic androgen R1881 (R1881; 5 nM), calcium (calcium chloride; 1 mM and 3 mM), the antiandrogens hydroxyflutamide (HF; 10 *μ*M), bicalutamide (BIC; 10 *μ*M), and enzalutamide (ENZ; 10 *μ*M).

### 2.2. Transient Transfection Assay

HEK293T cells were maintained in DMEM (Corning 10013CV) with 10% FBS. For transfection, the cells were plated at approximately 60 to 70% confluence in 6-well plates overnight in phenol red-free, lipoic acid-free IMEM supplemented with 5% charcoal-stripped serum (Valley Biomedical). The cells were then transfected with the AR plasmid for 24 hours using TransIT-LT1 reagent according to the manufacturer's (Mirus Bio) protocol. The cells were treated for 24 hours, and RNA was isolated.

### 2.3. RNA Isolation

After treatment, the media were aspirated, the cells were washed, and RNA was isolated using the TRIzol reagent (Ambion; Life Technologies) following the manufacturer's protocols as described elsewhere [16]. Briefly, the cells were resuspended in TRIzol, and chloroform was added. The samples were mixed, incubated for 3 minutes at room temperature, and centrifuged at 12,000 rpm for 30 minutes at 4°C, and the aqueous phase was collected. RNA was isolated with isopropanol. The 260 : 280 ratio and concentration were determined using the Nanodrop spectrophotometer (ND-1000).

### 2.4. Reverse Transcription (cDNA Synthesis)

For the reverse transcriptase reaction, 1 *μ*g of RNA was used to synthesize cDNA. First, RNA was treated with DNase 1 (DNase I amplification grade 100 U; Invitrogen) and incubated for 15 minutes at room temperature. EDTA was added, and the samples were incubated for 10 minutes at 65°C. For the reverse transcription step, each reaction contained 10 *μ*l DNase-treated RNA, 4.25 *µ*l molecular biology grade water, 10 *µ*l of Taq polymerase (M-MLV RT 5X Buffer), 11 *µ*l of 25 mM MgCl_2_ (Bioline), 10 *µ*l dNTP mix, 2.5 *µ*l random hexamer (50 *µ*M at 100 *µ*l; Invitrogen), 1 *µ*l RNase inhibitor (2000u 20U/*µ*l), and 1.25 *µ*l reverse transcriptase (Multiscribe). The mixture was incubated in a thermal cycler (MJ Research, Peltier Thermal Cycler PTC-225) for 10 minutes at 25°C, 30 minutes at 48°C, and 5 minutes at 95°C.

### 2.5. Quantitative Real-Time Reverse Transcription PCR

For the qRT-PCR assay, the master mix was made by using SensiMix II Probe Kit (Bioline) and the probes were TaqMan Assay on Demand (Applied Biosystems, Thermo Fisher Scientific). Each 10 *µ*L reaction contained 5.0 *µ*L universal master mix, 0.5 *µ*L of 20x Assay on Demand, and 4.5 *µ*L cDNA. The genes used to measure the expression of the calcium channels were calcium voltage-gated channel subunit alpha1 C (CACNA1-C; Applied Biosystems 4331182; assay ID: Hs00167681_m1), calcium voltage-gated channel subunit alpha1 D (CACNA1-D; Applied Biosystems 4331182; assay ID: Hs00167753_m1), calcium voltage-gated channel subunit alpha1 G (CACNA1-G; Applied Biosystems 4331182; assay ID: Hs00367969_m1), calcium voltage-gated channel subunit alpha1 H (CACNA1-H; Applied Biosystems 4331182; assay ID: Hs01103527_m1), transient receptor potential cation channel subfamily melastatin member 7 (TRPM-7; Applied Biosystems 4331182; assay ID: Hs00559080_m1), and transient receptor potential cation channel subfamily vanilloid member 6 (TRPV-6; Applied Biosystems 4331182; assay ID: Hs00536497_m1). The genes to measure the effect of hormone and calcium treatments were androgen receptor (*AR*; Applied Biosystems 4331182; assay ID: Hs00171172_m1), NK3 homeobox 1 (*NKX3.1.1*; Applied Biosystems 4331182; assay ID: Hs00171834_m1), transmembrane protease serine 2 (*TMPRSS2*; Applied Biosystems 4331182; assay ID: Hs01120965_m1), wingless-type MMTV integration site family, member 7B (*WNT7B*; Applied Biosystems 4331182; assay ID: Hs00536497_m1), FK506-binding protein (*FKBP5*; Applied Biosystems 4331182; assay ID: Hs01561006_m1), prostate-specific antigen (*PSA*; Applied Biosystems 4331182; assay ID: Dm01832137_m1), and 18S ribosomal subunit (*18S*; Applied Biosystems 4331182; assay ID: Hs99999901_s1). 18S was the endogenous control because it is not regulated by androgens. The samples were run in 7900 HT Real-Time PCR System. The ^ΔΔ^CT method (SDS 2.2 software) was used to evaluate gene expression.

### 2.6. Cell Growth Assay

For the cell growth assay, 5000 cells were plated into 96 wells with RPMI containing 10% FBS for 24 hours. The medium was changed to DMEM containing 10% FBS for 48 hours and then changed to lipoic-acid-free IMEM (Crystalgen or VitaScientific) containing 5% CSS (Sigma-Aldrich) for 48 hours. The cells were then treated with 5 nM DHT or 1 mM or 3 mM calcium in the presence or absence of 10 *μ*M enzalutamide. Cell growth was continuously measured for 8 days using xCELLigence RTCA.

### 2.7. Statistical Analysis

Statistical analysis was performed using Prism GraphPad software 6.1 (La Jolla, CA). All data are presented as the mean ± SEM. Differences between treatment groups (DHT, R1881, calcium, HF, BIC, and ENZ) and the control (untreated group) were assessed using Student's *t*-test to compare two groups or nonparametric one-way ANOVA, followed by Dunnett's (multiple comparisons to the same control) *post hoc* test. Differences in calcium channel expression among prostate cancer cell lines were analyzed also using one-way ANOVA, followed by Dunnett's (multiple comparisons to the same control) *post hoc* test. The null hypothesis is that the treatments have the same expression as the control. Statistical significance was set at a *P* value < 0.05. The statistical analysis used to compare the calcium channels in cancer cells compared with the control was also one-way ANOVA. The *post hoc* test used was Dunnett's multiple comparisons. Statistical significance was set at a *P* value < 0.05.

## 3. Results

### 3.1. Effects of Calcium and First- and Second-Generation Antiandrogens on the Expression of Androgen Receptor-Regulated Genes in PWR-1E Cells

**Figure 1:**
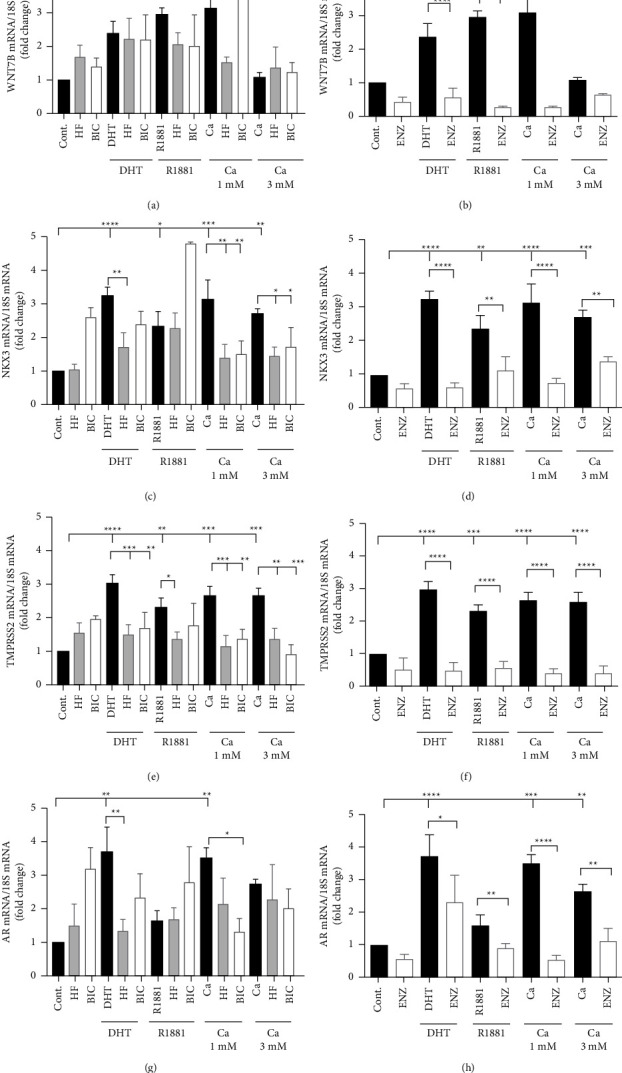
Effects of calcium and first- and second-generation antiandrogens on the expression of androgen receptor-regulated genes in PWR-1E cells. PWR-1E cells were maintained in hormone-free media and treated for 24 hours with dihydroxytestosterone (DHT; 5 nM), R1881 (5 nM), or calcium (Ca^+2^; 1 mM and 3 mM) in the absence and the presence of the first-generation antiandrogens hydroxyflutamide (HF; 10 *μ*M) and bicalutamide (BIC; 10 *μ*M) and the second-generation antiandrogen enzalutamide (ENZ; 10 *μ*M). RNA was isolated, and the amount of mRNA was measured using a qRT-PCR assay and normalized to the amount of 18S rRNA. Data are presented as fold change (mean ± SEM; biological replicates = 3; technical replicates = 3; *P* < 0.05; ^*∗*^*P* <  0.05, ^*∗∗*^*P* <  0.01, ^*∗∗∗*^*P* <  0.001, and ^*∗∗∗∗*^*P* <  0.0001). Effects of calcium on the expression of WNT7B (a, b), NKX3.1 (NKX3; (c, d)), TMPRSS2 (e, f), and AR (g, h) in the presence of first-generation antiandrogens hydroxyflutamide and bicalutamide or second-generation antiandrogen enzalutamide, respectively.

The PWR-1E cells were employed to ask whether calcium activates the androgen receptor and whether first- and second-generation antiandrogens block the effects of calcium in nontumorigenic prostate cells. The PWR-1E cells are immortalized human prostate cells derived from the normal prostate. The PWR-1E cells were maintained in hormone-free media and treated for 24 hours with dihydroxytestosterone (DHT; 5 nM), the synthetic androgen R1881 (5 nM), or calcium (1 or 3 mM) in the absence and the presence of the first-generation antiandrogens hydroxyflutamide (10 *μ*M) and bicalutamide (10 *μ*M) and the second-generation antiandrogen enzalutamide (10 *μ*M). In serum, the concentration of calcium ranges from 2.2 to 2.7 mM [17]. However, in hypocalcemia, the concentration is less than 2.12 mM [18], and in hypercalcemia, it is greater than 2.5 mM [19]. To mimic biologically relevant concentrations of serum calcium, cells were treated with 1 mM and 3 mM calcium. The ability of DHT, R1881, and calcium to increase the expression of the androgen-responsive genes WNT7B, NKX3.1, TMPRSS2, and the androgen receptor (AR) and the ability of the first- and second-generation antiandrogens to block this induction were measured (Figure 1). In the PWR-1E cells, DHT, R1881, and calcium increased the expression of WNT7B, NKX3.1, and TMPRSS2. The increase in expression was blocked by the second-generation antiandrogen enzalutamide but was not consistently blocked by the first-generation antiandrogens. In the case of WNT7B, treatment with DHT or R1881 resulted in an approximately 2.4- and 3.0-fold increase in expression, respectively, which was blocked by the second-generation antiandrogen enzalutamide but was not blocked by the first-generation antiandrogens hydroxyflutamide or bicalutamide (Figures 1(a) and 1(b)). Treatment with calcium also resulted in an approximately 3.1-fold increase in the expression of WNT7B that was blocked by enzalutamide and hydroxyflutamide but was not blocked by bicalutamide (Figures 1(a) and 1(b)). Treatment with DHT, R1881, or calcium (1 or 3 mM) also increased the expression of NKX3.1 by approximately 3.3-, 2.3-, 3.2- and 2.7-fold (Figures 1(c) and 1(d)) and TMPRSS2 by approximately 3.0-, 2.3-, 2.7-, and 2.7-fold (Figures 1(e) and 1(f)), respectively. The increase was blocked by enzalutamide but was not consistently blocked by the first-generation antiandrogens. Hydroxyflutamide blocked the effects of DHT, R1881, and calcium on the expression of TMPRSS2 and the effects of DHT and calcium on the expression of NKX3.1 but did not block the effect of R1881 on the expression of NKX3.1. Bicalutamide blocked the effects of DHT on the expression of TMPRSS2 and calcium on the expression of NKX3.1 and TMPRSS2 but did not block the effects of DHT on the expression of NKX3.1 or R1881 on the expression of NKX3.1 or TMPRSS2 (Figures 1(c)–1(f)). Androgens differentially regulate the expression of the androgen receptor. Androgens can downregulate the expression of the androgen receptor in many cells and tissues and upregulate expression in other cells and tissues [12, 20]. Treatment of PWR-1E cells with DHT resulted in an approximately 3.8-fold increase in the expression of the androgen receptor that was blocked by hydroxyflutamide and enzalutamide but not by bicalutamide. Similar to DHT, treatment with calcium (1 mM and 3 mM) increased the expression of the androgen receptor by approximately 3.5- and 2.7-fold that was inhibited by the enzalutamide but not consistently inhibited by bicalutamide (Figures 1(g) and 1(h)). Taken together, the results suggest that in normal prostate cells, calcium mimics the effects of androgens on gene expression and that the effects are blocked by the second-generation antiandrogen enzalutamide.

### 3.2. Effects of Calcium and Second-Generation Antiandrogen on the Expression of Androgen Receptor-Regulated Genes in HEK293T Cells Transiently Transfected with the Androgen Receptor

**Figure 2:**
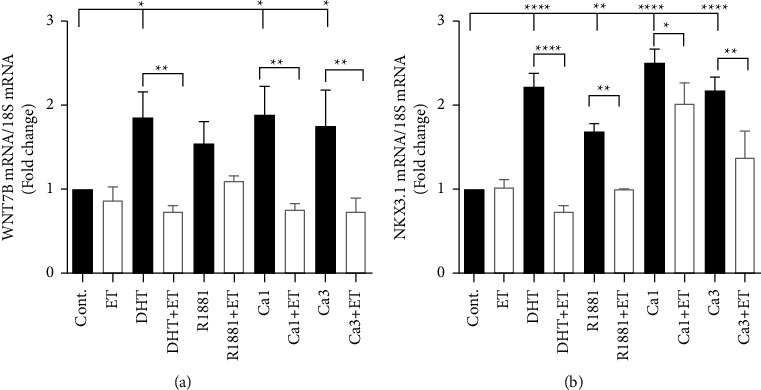
Effects of calcium in androgen receptor transfected HEK293T cells. HEK293T cells were plated in lipoic acid free and phenol red free IMEM containing 5% CCS for 24 hours and transiently transfected with the androgen receptor plasmid (2.5 *μ*g/*μ*l). After 24 hours, the medium was diluted to 1% CCS and the cells were treated with dihydroxytestosterone (DHT, 5 nM), R1881 (5 nM), or calcium (Ca_+2_, 1 mM and 3 mM) in the absence and the presence of the second-generation antiandrogen enzalutamide (ET, 10 *μ*M). RNA was isolated, and the amount of WNT7B (a) and NKX3.1 (b) mRNA was measured using a qRT-PCR assay and normalized to the amount of 18S rRNA. Data are presented as fold change (mean ± SEM; biological replicates = 3; technical replicates = 3; and *P* <  0.05, ^*∗*^*P* <  0.05, ^*∗∗*^*P* <  0.01, ^*∗∗∗*^*P* <  0.001, ^*∗∗∗∗*^*P* <  0.0001).

To demonstrate that calcium activates the androgen receptor and determine whether the second-generation antiandrogen blocks the effects of calcium, HEK293T cells that do not express the androgen receptor were transiently transfected with the androgen receptor, maintained in hormone-free media, and treated for 24 hours with dihydroxytestosterone (5 nM), R1881 (5 nM) or calcium (1 or 3 mM) in the absence and the presence of enzalutamide (10 *μ*M) (Figure 2). Treatment with DHT or calcium increased the expression of the endogenous genes WNT7B and NKX3.1. Treatment with DHT increased the expression of WNT7B and NKX3.1 by approximately 1.9- and 2.2-fold, respectively. Treatment with enzalutamide blocked the increase in WNT7B and NKX3.1. Similar to DHT, treatment with calcium increased the expression of WNT7B and NKX3.1 by approximately 1.8- to 2.8-fold and 2.1- to 2.5-fold, respectively, and treatment with enzalutamide blocked the effects of calcium, suggesting that calcium activates the androgen receptor transiently expressed in HEK293T cells.

### 3.3. Effects of Calcium and First- and Second-Generation Antiandrogens on the Expression of Androgen Receptor-Regulated Genes in LNCaP Cells

**Figure 3:**
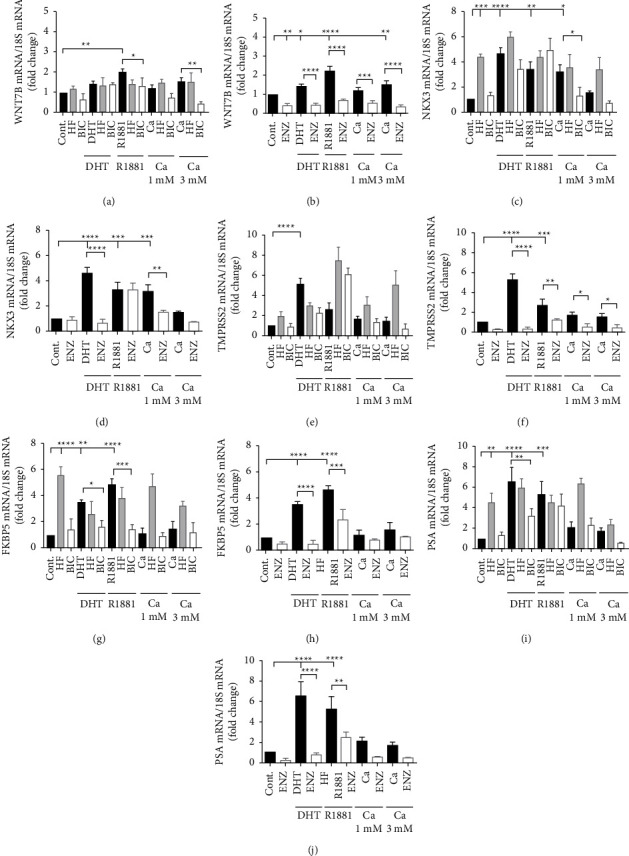
Effects of calcium and first- and second-generation antiandrogens on the expression of androgen receptor-regulated genes in LNCaP cells. LNCaP cells were maintained in hormone-free media and treated for 24 hours with dihydroxytestosterone (DHT; 5 nM), R1881 (5 nM), or calcium (Ca^+2^; 1 mM and 3 mM) in the absence and the presence of the first-generation antiandrogens Casodex (BIC; 10 *μ*M) or hydroxyflutamide (HF; 10 *μ*M) and the second-generation antiandrogen enzalutamide (ENZ; 10 *μ*M). RNA was isolated, and the amount of mRNA was measured using a qRT-PCR assay and normalized to the amount of 18S rRNA. Data are presented as fold change (mean ± SEM; biological replicates = 3; technical replicates = 3; ^*∗*^*P* <  0.05, ^*∗∗*^*P* <  0.01, ^*∗∗∗*^*P* <  0.001, and ^*∗∗∗∗*^*P* <  0.0001). Effects of calcium on the expression of WNT7B (a, b), NKX3.1 (NKX3; (c, d)), TMPRSS2 (e, f), FKBP5 (g, h), and PSA (i, j) in the presence of first-generation antiandrogens hydroxyflutamide and bicalutamide and the second-generation antiandrogen enzalutamide, respectively.

Treatment-induced mutations in the ligand-binding domain of the androgen receptor are common and known to convert antiandrogens into agonists. The LNCaP prostate cancer cell line contains a T877A point mutation in the ligand-binding domain of the androgen receptor which converts hydroxyflutamide into an agonist [21]. In this study, LNCaP cells were maintained in hormone-free media and treated for 24 hours with dihydroxytestosterone (DHT; 5 nM), the synthetic androgen R1881 (5 nM), or calcium (1 or 3 mM) in the absence and the presence of the first-generation antiandrogens hydroxyflutamide (10 *μ*M) and bicalutamide (10 *μ*M) and the second-generation antiandrogen enzalutamide (10 *μ*M). A previous study showed that treatment with a calcium ionophore or an inhibitor of calcium ATPases to increase intracellular calcium reduced AR expression in LNCaP cells [22]. In this study, the ability of DHT, R1881, and calcium to increase the expression of the androgen-responsive genes WNT7B, NKX3.1, TMPRSS2, FKBP5, and PSA and the ability of the first- and second-generation antiandrogens to block the induction were measured (Figure 3). As expected, treatment of LNCaP cells with hydroxyflutamide increased the expression of NKX3.1, FKBP5, and PSA (Figures 3(c), 3(d), 3(g)–3(j)) but had no effect on the expression of WNT7B and TMPRSS2 (Figures 3(a), 3(b), 3(e), 3(f)). Treatment with R1881 increased the expression of WNT7B, NKX3.1, TMPRSS2, FKBP5, and PSA by approximately 2.3-, 3.3-, 2.7-, 4.7-, and 5.3-fold, respectively (Figures 3(a)–3(j)). Bicalutamide blocked the increase in expression of WNT7B and FKBP5 (Figures 3(a), 3(b), 3(g) and 3(h)) but did not block the increase in expression of NKX3.1, TMPRSS2, and PSA (Figures 3(c)–3(f), 3(i) and 3(j)). On the other hand, enzalutamide blocked the increase in expression of WNT7B, TMPRSS2, FKBP5, and PSA (Figures 3(a), 3(b), 3(e)–3(j)) but did not block the increase in expression of NKX3.1 (Figures 3(c) and 3(d)). Treatment with calcium also resulted in an approximately 3-fold increase in the expression of NKX3.1 which was blocked by bicalutamide and enzalutamide (Figures 3(c) and 3(d)). Taken together, the results suggest that calcium activates the AR containing a T877A mutation and that activation is more effectively blocked by the second-generation antiandrogen.

### 3.4. Expression of Calcium Channels in Prostate Cell Lines

**Figure 4:**
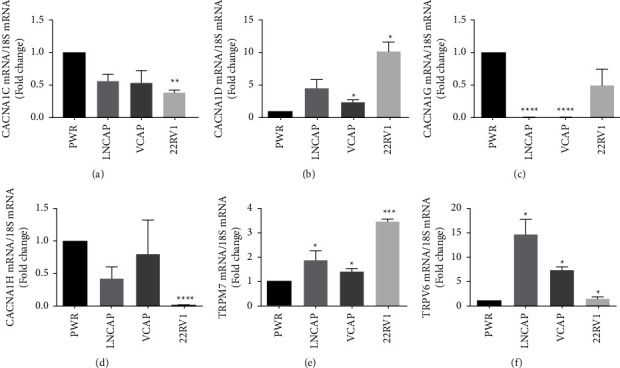
Calcium channels in hormone-independent and castration-resistant prostate cancer cells. PWR-1E (PWR), LNCaP, VCaP, and 22RV1 cells were plated in improved minimum essential media (IMEM) containing phenol red and 10% FBS serum. To determine the expression of calcium channels, the amount of CACNA1-C, -D, -G, and -H; TRPM-7; and TRPV-6 mRNA was measured using a quantitative real-time PCR assay and normalized to the amount of 18S rRNA. Data are presented as fold change compared to PWR cells (mean ± SEM; biological replicates = 3; technical replicates = 3; ^*∗*^*P* <  0.05, ^*∗∗*^*P* <  0.01, ^*∗∗∗*^*P* <  0.001, and ^*∗∗∗∗*^*P* <  0.0001).

Dysregulation of calcium channels contributes to many cellular processes including proliferation, differentiation, and apoptosis. The ability of calcium to activate the androgen receptor in the absence of androgen suggests that calcium channels may also play a role in castration resistance. To address this question, the expression of calcium channels CACNA1-C, -D, -G, and -H, which are activated by membrane potential and the transient receptor potential (TRP) channels TRPM-7, which respond to magnesium and TRPV-6 which is constitutively active, was measured in the normal PWR-1E prostate cells, the hormone-responsive LNCaP prostate cancer cells, and the castration-resistant VCaP and 22RV1 prostate cancer cells (Figure 4). Compared to normal prostate cells, the hormone-responsive and hormone-resistant prostate cancer cells overexpress CACNA1-D, TRPM-7, and TRPV-6. There was an approximately 5-fold increase in CACNA1-D and an approximately 15-fold increase in TRPV-6 in the hormone-responsive LNCaP cells. In the castration-resistant VCaP cells, there was an approximately 3-fold increase in CACNA1-D and an approximately 6-fold increase in TRPV-6. In the 22RV1 cells, there was an approximately 10-fold increase in CACNA1-D and an approximately 3.5-fold increase in TRPM-7. Taken together, the results suggest that calcium channels are differentially expressed in prostate cancer cells.

### 3.5. Effects of Calcium on the Growth of LNCaP Cells

**Figure 5:**
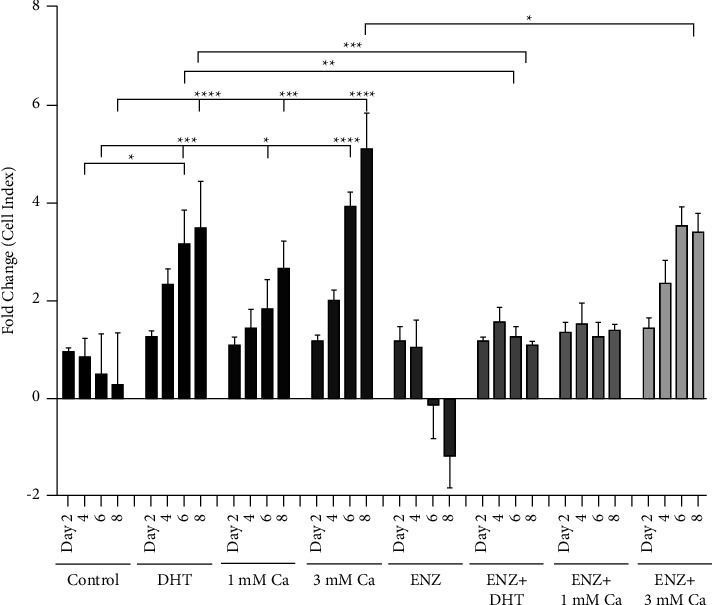
Effects of calcium on the growth of LNCaP cells. LNCaP cells were maintained in hormone-free media and treated with dihydroxytestosterone (DHT; 5 nM) or calcium (Ca; 1 mM and 3 mM) in the absence and the presence of the antiandrogen enzalutamide (ENZ; 10 *μ*M) and continuously monitored for 8 days. Data are presented as fold change on days 2, 4, 6, and 8 (mean ± SEM; biological replicates = 3; technical replicates = 3; *P* < 0.05; ^*∗*^*P* <  0.05, ^*∗∗*^*P* <  0.01, ^*∗∗∗*^*P* <  0.001, and ^*∗∗∗∗*^*P* <  0.0001).

To ask whether calcium mimics the effects of androgens on growth, LNCaP cells were treated with DHT (5 nM) or calcium (1 or 3 mM) in the presence and absence of the enzalutamide and monitored for 8 days (Figure 5). As expected, treatment with DHT resulted in an approximately 3.5-fold increase in the cell number that was blocked by the antiandrogen. Treatment with calcium (1.0 and 3.0 mM) resulted in an approximately 2.7- and 5.2-fold increase, respectively, which was blocked by the antiandrogen.

## 4. Discussion

First-line standard of care for advanced and metastatic prostate cancer is androgen deprivation therapy which includes surgical castration, medical castration, antiandrogens, and inhibition of androgen synthesis. Although still dependent on the androgen/androgen receptor axis, most prostate cancers progress to a castration-resistant phenotype that is due, in part, to intratumoral and adrenal synthesis of androgens, overexpression and mutation of the androgen receptor, expression of AR splice variants, stabilization of AR by chaperones, and androgen-independent activation of the receptor by growth factors and intracellular signal transduction pathways [23, 24]. In addition to alterations in the androgen/androgen receptor axis, the progression of the disease is associated with loss of calcium homeostasis due, in part, to the involvement of calcium in cell survival, proliferation, motility and invasion, metastasis, and cell death [25]. The results presented in this study show that calcium activates the androgen receptor in nontumorigenic and tumorigenic prostate cells, second-generation antiandrogens are more effective than first-generation antiandrogens in blocking calcium activation of AR, and calcium channels are overexpressed in hormone-dependent and hormone-resistant prostate cancer cells. Taken together, the results suggest a novel role of calcium in prostate cancer.

The androgen receptor is a member of the nuclear receptor superfamily that is a ligand inducible transcription factor. Similar to other members of the superfamily, the androgen receptor is composed of the N-terminal domain that contains activation function-1 (AF-1), the central DNA-binding domain, the hinge region, and the C-terminal domain that contains the ligand-binding domain (LBD). The binding of the ligand to the LBD results in the formation of activation function-2 (AF-2). We have previously shown that cadmium, a bivalent cation that mimics calcium, activates AR in the absence of hormone through a high-affinity interaction with the LBD [12]. The ligand-binding domain of nuclear receptors contains 12 *α* helices (AR is missing helix H2) that form a sandwich structure of three layers of antiparallel helices that are flanked by helix H12 [26, 27]. Located in the central core of the ligand-binding domain is the ligand-binding pocket. Based on the crystal structure of the retinoid X receptor in the absence and presence of its ligand, it is thought that several major conformational changes occur in the LBD as a result of ligand binding in the pocket [27]. In the absence of ligand, helix H12 flanks the ligand-binding pocket. The binding of the ligand induces several conformational changes that result in the repositioning of helix H11 to create the dimerization domain and the repositioning of helix H12 over the ligand-binding pocket. Helix H12 along with helices H3 and H4 then creates the AF-2 domain, a coactivator-binding site [28–31]. We have also shown that calcium and cadmium activate estrogen receptor-*α* (ER*α*) through the LBD in the absence of estradiol and that calcium is a physiological ligand of ER*α* [9–11]. In contrast to estradiol that binds inside the ligand-binding pocket, calcium binds to four sites on the aqueous surface of the ligand-binding domain [9]. The four sites include a site on the N-terminal end of helix H12, the C-terminal end of helix H11, the interface of helices H10/H11, and between helices H4 and H12. In proteins, metals such as calcium have several functions including the formation and stabilization of protein structures through interactions with different amino acids. Although the mechanism by which calcium activates ER*α* is not completely understood, the identification of calcium-binding sites in the LBD suggests that, similar to estradiol, calcium promotes a conformational change that results in the repositioning of helix H12 and the formation of the AF-2 domain. The structural and amino acid homology between the ligand-binding domains of ER*α* and AR also suggests that calcium activates the androgen receptor by interacting with and promoting a conformational change in the LBD of the receptor. Antiandrogens compete with androgens for binding in the ligand-binding pocket. However, the precise mechanism by which antiandrogens inhibit transcription is not clear as the ligand-binding domain of the AR has only been crystalized with the agonists, testosterone, dihydroxytestosterone, and R1881, and with nonsteroidal tissue-specific agonists [30, 31]. Based on the crystal structure of other nuclear receptors complexed with their antagonists, it is thought that antiandrogens compete with androgens for binding in the pocket, displacing helix H12, and distorting the AF-2 domain. In contrast to competing with androgens, enzalutamide appears to be a noncompetitive inhibitor of calcium. The ability of enzalutamide to inhibit calcium activation of AR suggests that it may also alter/distort the calcium interaction sites located on helices H4, H10, and H11. However, we cannot rule out the possibility that enzalutamide inhibits calcium activation of AR by inhibiting nuclear translocation and binding to DNA. The mechanism by which calcium activates AR and enzalutamide inhibits activation remains to be determined.

In cells, there are two main sources of calcium: release from intracellular organelles such as the endoplasmic reticulum and mitochondria and influx from the extracellular environment. Growth factors and cytokines that signal through the phospholipase C (PLC)-inositol trisphosphate (IP3) pathway release calcium from the endoplasmic reticulum, whereas membrane channels increase the influx of calcium from the extracellular environment. Increasing evidence links loss of calcium homeostasis in prostate cells and prostate cancer with alterations in the IP3 receptor in the endoplasmic reticulum and the T-type calcium channels, store-operated calcium channels Orai1 and STIM1, and the TRP channels TRPC1/C4, TRPM-8, and TRPV-6 in the plasma membrane [25, 32–35]. We have previously shown that in hormone-dependent breast cancer cells, epidermal growth factor (EGF) activates the PLC*γ*-IP3 pathway and increases the concentration of intracellular calcium which in turn interacts with and activates ER*α* [9]. We have also shown that hormone-independent and hormone-resistant breast cancer cells overexpress calcium channels, have high concentrations of intracellular calcium, and overexpress estrogen-responsive genes. Treatment of hormone-independent cells with calcium channel blockers decreases the concentration of intracellular calcium and mimics the effects of an antiestrogen on the expression of estrogen-responsive genes and cell growth, while treatment of hormone-resistant cells with a calcium channel blocker and an antiestrogen reversed resistance to the antiestrogen [16]. In this study, we measured the expression of the L-type and T-type voltage-gated calcium channels, the nonselective cation channels TRPM-6 and TRPM-7, and the selective calcium channel TRPV-6 in nontumorigenic prostate cells and hormone-dependent and hormone-resistant prostate cancer cells. Compared to nontumorigenic cells, the hormone-dependent cells overexpressed TRPM-7 and TRPV-6 and the hormone-resistant cells overexpressed CACNA1-D, TRPM-7, and TRPV-6, supporting the role of calcium channels in prostate cancer.

There is also evidence linking calcium intake with prostate cancer. However, the association between calcium intake and prostate cancer is not well understood and sometimes contradictory (reviewed in [36]). Several dietary studies show an association between high intake of calcium and a higher risk of developing prostate cancer, while other studies show an association between high intake of dairy, but not nondairy, calcium, and a higher risk of developing prostate cancer [37–40]. Although nondairy calcium does not appear to be associated with a higher risk of developing prostate cancer, it is associated with fatal prostate cancer [41, 42]. A small number of studies also show an association between high intake of whole milk and a higher risk of prostate cancer recurrence and prostate cancer-specific death [40, 43]. However, other dietary studies show no association between intake of calcium and risk of prostate cancer [41, 44, 45].

In summary, treatment of AR-positive prostate cells with calcium increased the transcripts of androgen-responsive genes that was blocked by the second-generation antiandrogen enzalutamide but not consistently blocked by the first-generation antiandrogens. Although the study did not measure the binding of calcium to the LBD of AR and the binding of AR to its response element, we previously showed that cadmium, which mimics calcium, binds with high affinity to the LBD of AR and increases gene expression through an androgen response element [12]. We have also shown that calcium and cadmium bind to the LBD of ER*α* and increase gene expression by calcium through an estrogen response element [9, 11]. The increase in the transcripts of androgen-responsive genes and the ability of an antiandrogen to block the increase demonstrate that calcium activates the androgen receptor to increase the transcription of AR-regulated genes. Importantly, treatment with calcium increased androgen receptor-mediated cell proliferation that was blocked by an antiandrogen. Compared to normal cells, several calcium channels are overexpressed in hormone-responsive and castration-resistant prostate cancer cells, suggesting a role of calcium channels in the disease. The ability of calcium to activate the androgen receptor and the association of calcium intake and calcium channels with prostate cancer suggest that new therapies for the treatment of prostate cancer, which include drugs that target specific calcium channels or transporters, should be further investigated.

## Data Availability

Data are available upon request.
